# Beyond Resistance: Carbapenemase Gene Profiles and Mortality in ICU Patients with Carbapenem-Resistant *Klebsiella pneumoniae* Infections

**DOI:** 10.3390/ijms27209388

**Published:** 2026-10-21

**Authors:** Esra Kaya, Elif Okumuş, Elif Gülsüm Torun

**Affiliations:** 1 Department of Medical Microbiology, Kahramanmaraş Necip Fazıl City Hospital, Kahramanmaraş 46050, Türkiye; elifgulsumtorun@gmail.com; 2 Department of Medical Microbiology, Van Training and Research Hospital, Van 65300, Türkiye; drelifokumus@gmail.com

**Keywords:** carbapenem-resistant *Klebsiella pneumoniae*, carbapenemase genes, OXA-48, NDM, CTX-M, intensive care unit, mortality, molecular epidemiology

## Abstract

Carbapenem-resistant *Klebsiella pneumoniae* (CRKP) causes serious infections in intensive care units (ICUs), but the relationship between carbapenemase gene profiles and mortality remains uncertain. This prospective two-center cohort included 94 adult ICU patients with clinical CRKP infection from April to September 2026. The first clinical isolate from each patient was included. *bla*_KPC_, *bla*_NDM_, *bla*_VIM_, *bla*_IMP_, *bla*_OXA-48_, and *bla*_CTX-M_ genes were investigated by polymerase chain reaction. The primary outcome was 28-day ICU mortality; 14-day and final ICU mortality were secondary outcomes. *bla*_OXA-48_, *bla*_NDM_, *bla*_KPC_, and *bla*_CTX-M_ were detected in 65 (69.1%), 49 (52.1%), 19 (20.2%), and 73 (77.7%) of isolates, respectively; *bla*_NDM_ and *bla*_OXA-48_ co-occurred in 39 (41.5%). Fourteen-day, 28-day, and final ICU mortality rates occured in 38 (40.4%), 48 (51.1%), and 53 (56.4%), respectively. No statistically significant associations were observed between individual genes, the presence of two carbapenemase genes, or carbapenemase profiles and any mortality endpoint; adjusted analyses of 28-day ICU mortality yielded similar findings. In the exploratory Firth model, the profile effect was not significant (*p* = 0.198), whereas a higher age-adjusted Charlson Comorbidity Index and bloodstream infection or ventilator-associated pneumonia, compared with urinary tract infection, were associated with higher odds of 28-day ICU mortality. Frequent gene co-occurrence was observed; however, clinically meaningful gene–mortality associations cannot be excluded because of imprecise estimates.

## 1. Introduction

*Klebsiella pneumoniae* is a major cause of serious healthcare-associated infections, including bloodstream infection, urinary tract infection, and ventilator-associated pneumonia, particularly in intensive care units (ICUs). The spread of carbapenem resistance represents an important global public health problem because it limits treatment options and is associated with high mortality in severe infections. In the World Health Organization’s 2024 Bacterial Priority Pathogens List, carbapenem-resistant Enterobacterales were classified as critical-priority pathogens, and carbapenem-resistant *K. pneumoniae* (CRKP) received the highest overall score (84%) among the pathogen-resistance combinations evaluated [1]. In a recent study from a tertiary hospital in Türkiye, the overall prevalence of CRKP among *K. pneumoniae* isolates was 66.8%, and 62.5% of CRKP-positive samples originated from ICU patients [2]. Critical illness, prolonged hospitalization, invasive procedures, and exposure to broad-spectrum antibiotics further increase the clinical importance of CRKP infections in ICU patients [3,4]. Pooled estimates have also indicated a CRKP colonization incidence of 22.3%, with most of the contributing studies involving ICU populations [4].

Carbapenem resistance in *K. pneumoniae* is primarily mediated by carbapenemase production, although extended-spectrum beta-lactamase (ESBL) or AmpC production combined with alterations in outer-membrane porins may also contribute [3,4,5]. The most clinically important carbapenemases include class A KPC enzymes, class B metallo-beta-lactamases such as NDM, VIM, and IMP, and class D OXA-48 enzymes. Their distribution varies geographically. OXA-48, first identified in Türkiye, remains one of the predominant carbapenemases in Türkiye and neighboring regions, whereas NDM and isolates co-harboring OXA-48 and NDM have increasingly been reported in recent years [4,6,7]. Carriage of carbapenemase genes on mobile genetic elements facilitates horizontal transfer between bacteria, while the spread of carbapenemase-producing high-risk clones contributes to transmission within and between healthcare institutions [3,5]. Molecular identification of these genes is therefore important not only for characterizing resistance mechanisms but also for epidemiological surveillance and infection control [3,5].

Although CTX-M-type ESBLs are not carbapenemases, their co-occurrence with carbapenemase genes may contribute to a broader beta-lactam resistance profile [4,5]. However, the clinical importance of specific carbapenemase genes or the co-occurrence of multiple resistance genes for patient outcomes remains incompletely understood. Mortality among patients with CRKP infection may be influenced not only by the resistance mechanism but also by disease severity, comorbidities, the site of infection, septic shock, appropriate antimicrobial therapy, and the clonal or virulence characteristics of the isolate [5,6,7,8,9]. Most previous outcome studies have focused on bloodstream infections or a single carbapenemase type. Prospective evidence jointly evaluating individual resistance genes, carbapenemase gene burden, and mutually exclusive carbapenemase profiles across different ICU infection syndromes and prespecified early and final ICU mortality outcomes remains limited. This two-center study aimed to characterize the molecular epidemiology of CRKP clinical infections in adult ICU patients and to explore whether resistance-gene patterns were associated with ICU mortality. Specifically, the distribution and co-occurrence of *bla*_KPC_, *bla*_NDM_, *bla*_VIM_, *bla*_IMP_, *bla*_OXA-48_, and *bla*_CTX-M_ were described using the first clinical isolate obtained from each patient. The primary analysis evaluated the associations of individual resistance genes, the number of carbapenemase genes, and mutually exclusive carbapenemase profiles with 28-day ICU mortality. Secondary analyses evaluated the associations of the same molecular characteristics with 14-day ICU mortality and final ICU mortality.

## 2. Results

### 2.1. Patient Characteristics and Clinical Outcomes

A total of 94 patients were included. Thirty-eight patients (40.4%) died in the ICU within 14 days after the index culture, 48 (51.1%) died in the ICU within 28 days, and 53 (56.4%) died during their ICU stay. The median age of the study population was 70 years (IQR, 58–78), and 59 patients (62.8%) were male. Non-survivors had higher age-adjusted Charlson Comorbidity Index (CCI) scores than survivors [median, 7 (IQR, 6–9) vs. 5 (IQR, 3–6); *p* < 0.001].

The distributions of specimen and infection types differed according to final ICU outcome (*p* = 0.010). Final ICU mortality was 73.3% (22/30) among patients with CRKP-positive blood cultures and bloodstream infection and 35.5% (11/31) among those with CRKP-positive urine cultures and urinary tract infection. The interval from the first CRKP-positive culture to the end of the ICU stay was shorter among non-survivors than among survivors [median, 8 (IQR, 5–17) vs. 14 (IQR, 6–45) days; *p* = 0.011]. No significant between-group differences were demonstrated in age, sex, study center, total ICU length of stay, or time from ICU admission to the first CRKP-positive culture (Table 1).

### 2.2. Distribution of Resistance Genes and Molecular Profiles

Among the 94 CRKP isolates examined, *bla*_OXA-48_ was detected in 65 (69.1%), *bla*_NDM_ in 49 (52.1%), and *bla*_KPC_ in 19 (20.2%). *bla*_VIM_ and *bla*_IMP_ were not detected in any isolate. *bla*_CTX-M_ was detected in 73 isolates (77.7%). All isolates carried at least one carbapenemase gene.

A single carbapenemase gene was detected in 55 isolates (58.5%), whereas two carbapenemase genes were detected in 39 (41.5%). The most frequent carbapenemase profile was *bla*_NDM_ + *bla*_OXA-48_ [39/94 (41.5%)], and the most frequent complete resistance-gene profile including *bla*_CTX-M_ was *bla*_NDM_ + *bla*_OXA-48_ + *bla*_CTX-M_ [37/94 (39.4%)]. Detailed distributions are presented in Table 2, and gene co-occurrence patterns are shown in Figure 1.

### 2.3. Unadjusted Associations with ICU Mortality

No significant associations were demonstrated between the presence of *bla*_KPC_, *bla*_NDM_, *bla*_OXA-48_, and *bla*_CTX-M_ and 14-day ICU, 28-day ICU, or final ICU mortality. Similarly, no significant differences in any mortality outcome were demonstrated between patients whose isolates carried one versus two carbapenemase genes or among the mutually exclusive carbapenemase profiles (Table 3 and Table 4).

### 2.4. Multivariable Analysis of 28-Day ICU Mortality

After adjustment for age-adjusted CCI, infection type, and study center, no statistically significant associations were demonstrated between 28-day ICU mortality and the presence of *bla*_KPC_, *bla*_NDM_, *bla*_OXA-48_, or *bla*_CTX-M_ or the presence of two carbapenemase genes (Table 5 and Figure 2). However, the wide confidence intervals for some molecular variables did not exclude clinically meaningful associations.

In the exploratory Firth penalized logistic regression analysis, the overall association between carbapenemase gene profile and 28-day ICU mortality was not statistically significant (*p* = 0.198). Compared with the *bla*_OXA-48_ alone profile, no clear association was demonstrated for the other profiles; the particularly wide confidence interval for the *bla*_NDM_ alone group indicated substantial uncertainty in the estimate. A sensitivity analysis using standard maximum likelihood estimation did not alter the direction or overall interpretation of the findings.

In the profile-based Firth model, each one-point increase in the CCI was associated with higher odds of 28-day ICU mortality (Firth-adjusted OR, 1.91; 95% CI, 1.47–2.67; *p* < 0.001). Compared with urinary tract infection, bloodstream infection (Firth-adjusted OR, 13.10; 95% CI, 3.00–72.58; *p* < 0.001) and ventilator-associated pneumonia (Firth-adjusted OR, 5.16; 95% CI, 1.31–24.25; *p* = 0.018) were associated with higher odds of 28-day ICU mortality. The Firth-adjusted odds ratio for Necip Fazıl City Hospital versus Van Training and Research Hospital was 0.29 (95% CI, 0.08–0.99; *p* = 0.048). Adjusted associations between molecular resistance characteristics and 28-day ICU mortality are presented in Table 5 and Figure 2.

### 2.5. Exploratory Comparisons Between Study Centers

Exploratory comparisons by study center are presented in Supplementary Table S1. Patients at Necip Fazıl City Hospital had a higher age-adjusted CCI, and urinary tract infections were more frequent at this center, whereas bloodstream infections and ventilator-associated pneumonia were more frequent at Van Training and Research Hospital. *bla*_OXA-48_, *bla*_CTX-M_, the presence of two carbapenemase genes, and the *bla*_NDM_ + *bla*_OXA-48_ profile were more frequent at the Van center, while *bla*_KPC_ was more frequent at Necip Fazıl City Hospital. Despite these clinical and molecular differences, no statistically significant between-center differences were demonstrated in *bla*_NDM_ frequency, ICU mortality outcomes, or ICU length-of-stay measures.

## 3. Discussion

In this study, carbapenemase genes and *bla*_CTX-M_ were investigated in CRKP strains isolated as causative agents of clinical infection from adult ICU patients at two centers, and the associations between these molecular characteristics and mortality were evaluated. The principal contribution of this study is its integrated evaluation of individual resistance genes, carbapenemase gene burden, and mutually exclusive carbapenemase profiles across three clinically defined ICU infection types and three ICU mortality outcomes, rather than restricting the analysis to a single infection source or carbapenemase type. *bla*_OXA-48_ was the most frequently detected carbapenemase gene, *bla*_NDM_ was detected in more than half of the isolates, and *bla*_NDM_ and *bla*_OXA-48_ co-occurred in a substantial proportion. *bla*_CTX-M_ was also highly prevalent. No statistically significant associations were demonstrated between 14-day, 28-day, or final ICU mortality and the individual presence of *bla*_KPC_, *bla*_NDM_, *bla*_OXA-48_, or *bla*_CTX-M_, the presence of two carbapenemase genes, or the identified carbapenemase profiles. Nevertheless, because the wide confidence intervals for some molecular estimates did not exclude clinically meaningful associations, these findings should not be interpreted as evidence of equivalence or definitive absence of association between molecular groups. Among the measured clinical variables, comorbidity burden and infection type showed clearer associations with 28-day ICU mortality than did the molecular variables.

The predominance of *bla*_OXA-48_ in our study is consistent with epidemiological data reported from Türkiye. In a multicenter prospective study conducted in an OXA-48-endemic setting in Türkiye, OXA-48-like carbapenemase alone was reported in 75% of carbapenem-resistant Klebsiella bloodstream infections, whereas OXA-48-like enzymes co-occurred with NDM in 16% [10]. Kurt et al. detected *bla*_OXA-48_ in 74.8% of CRKP bacteremia cases, whereas *bla*_NDM_, *bla*_KPC_, and *bla*_OXA-48_-*bla*_NDM_ co-occurrence were found at lower frequencies [6]. OXA-48 was also the most frequently detected carbapenemase among carbapenem-resistant *K. pneumoniae* blood isolates collected from different centers in Türkiye by Zarakolu et al., followed by NDM and KPC [11]. A more recent ICU study reported high frequencies of *bla*_NDM_ and *bla*_OXA-48_-*bla*_NDM_ co-occurrence in addition to *bla*_OXA-48_ [7]. The predominance of *bla*_OXA-48_ and the high frequency of *bla*_NDM_ in our study suggest that *bla*_OXA-48_ remains important in Türkiye, while *bla*_NDM_ and isolates co-harboring the two genes are becoming increasingly prominent.

The high frequency of isolates co-harboring *bla*_NDM_ and *bla*_OXA-48_ in our study is not unique to Türkiye. A study conducted in Romania reported that CRKP isolates co-producing NDM and OXA-48-like carbapenemases were common [12]. Similarly, in a study of carbapenem-resistant Enterobacterales bloodstream infections in Thailand, *bla*_NDM-1_ and *bla*_OXA-48_ co-occurred in 51.7% of the isolates that underwent molecular testing [13]. In contrast, IMP-type carbapenemases predominated in a series from Japan, whereas OXA-48 and NDM were less frequent [14]. These differences show that carbapenemase epidemiology can vary substantially between countries, regions, and even hospitals within the same country. The ability of OXA-48-like carbapenemases to spread rapidly through different *K. pneumoniae* clones and mobile genetic elements also contributes to this regional diversity [15].

In our study, *bla*_CTX-M_ co-occurred with at least one carbapenemase gene in most isolates. *bla*_CTX-M_ is not a carbapenemase and was therefore not included in the carbapenemase gene count. Nevertheless, its co-occurrence with carbapenemases indicates that the isolates possessed a broader beta-lactam resistance repertoire and that different resistance mechanisms can accumulate within the same bacterium. In the genomic analysis of CRKP isolates from bloodstream and pneumonia specimens by Cox et al., extended-spectrum beta-lactamase genes also commonly co-occurred with carbapenemase genes [5]. The ability of plasmids carrying *bla*_OXA-48_-like genes to disseminate together with other resistance genes offers another possible explanation for this pattern [15]. No statistically significant association was demonstrated between *bla*_CTX-M_ and mortality in our study. However, because the wide confidence interval around the adjusted estimate did not exclude a clinically meaningful association, no definitive conclusion can be drawn regarding the relationship between *bla*_CTX-M_ and mortality.

One of the principal findings of this study was that no statistically significant associations were demonstrated between the investigated genes or gene combinations and mortality after adjustment for the measured confounders. However, with 94 patients and small numbers in several molecular-profile subgroups, statistical power and the precision of the estimates were limited. The wide confidence intervals in Table 5, particularly for the *bla*_NDM_-alone profile (adjusted OR, 2.73; 95% CI, 0.37–21.54), encompassed both potentially protective and harmful associations; therefore, clinically meaningful molecular effects cannot be excluded. The wide confidence interval for bloodstream infection (adjusted OR, 13.10; 95% CI, 3.00–72.58) likewise indicates that, although an association was demonstrated, its magnitude remains uncertain. Although Firth penalized logistic regression was used to reduce small-sample and separation bias, this method cannot compensate for the limited information available in sparse subgroups. Accordingly, the profile-specific estimates should be regarded as exploratory and hypothesis-generating rather than as evidence of equivalence or absence of association. A multicenter prospective study from Türkiye reported that 30-day mortality was more strongly associated with patient-related clinical characteristics, such as SOFA score and immunosuppression, than with carbapenemase type [10]. A recent study from Türkiye that directly evaluated *bla*_OXA-48_ and *bla*_NDM_ co-occurrence also considered molecular findings alongside clinical characteristics and noted that mortality could not be explained solely by carbapenemase co-occurrence [7]. Conversely, some studies have reported differences in clinical outcomes between carbapenemase types. In the Italian ALARICO study, bloodstream infections caused by metallo-beta-lactamase-producing Enterobacterales were associated with higher attributable mortality than those caused by KPC-producing isolates [16]. Pudpong et al. showed that 14-day mortality may be higher in some treatment groups with infections caused by isolates harboring *bla*_NDM-1_ or co-harboring *bla*_NDM-1_ and *bla*_OXA-48_ [17]. The study from Thailand also reported that clinical factors, including sepsis or septic shock, neutropenia, and the treatment administered, influenced mortality in carbapenem-resistant Enterobacterales (CRE) bloodstream infections involving *bla*_NDM-1_ and *bla*_OXA-48_ co-occurrence [13]. Differences among studies may reflect variation in patient populations, infection sources, bacterial species, carbapenemase subtypes, and antimicrobial treatments. Moreover, the clinical impact of a particular carbapenemase may be related less to the presence of the enzyme itself than to the activity of the available therapeutic agents against it.

Another reason why mortality cannot be explained solely by the name of the carbapenemase gene is that isolates carrying the same gene may differ in clonal background, virulence, and accompanying resistance mechanisms. A study of a KPC-2-producing sequence type 16 clone reported very high mortality rates and demonstrated that a particular clonal background may contribute to clinical outcomes independently of the carbapenemase gene [9]. Similarly, a prospective genomic study of CRKP bloodstream infections across two time periods showed that clones and resistance genes may change over time [18]. These findings indicate that clinical outcomes cannot be reduced to the presence of *bla*_KPC_, *bla*_NDM_, or *bla*_OXA-48_ alone; carbapenemase subtype, bacterial clonal background, virulence characteristics, accompanying resistance genes, and treatment should be considered together.

Among the clinical variables measured in our study, age-adjusted CCI and infection type showed clear associations with 28-day ICU mortality. The association between increasing comorbidity burden and mortality highlights the importance of underlying diseases and physiological reserve in the response of critically ill patients to infection. The higher odds of mortality observed with bloodstream infection and ventilator-associated pneumonia than with urinary tract infection may reflect the greater severity of systemic illness accompanying these infections. Previous studies have identified septic shock, high disease-severity scores, organ failure, immunosuppression, and inappropriate initial antimicrobial therapy as major clinical factors associated with mortality in CRKP infections [8,10,13,19]. However, acute disease-severity measures, such as SOFA score and septic shock, and patient-level data on the appropriateness and timing of antimicrobial therapy were not systematically collected as study variables and could not be reconstructed consistently across the entire two-center cohort. Restricting the analysis to one center or to selected patients with available data could have introduced selection bias and would have further reduced statistical precision. Therefore, these variables could not be reliably incorporated into the multivariable models; residual confounding remains possible, and the adjusted molecular estimates should not be interpreted as independent causal effects.

The differences in the distributions of carbapenemase genes and profiles between the two study centers were also noteworthy. Nevertheless, no clear difference in unadjusted mortality rates was demonstrated between centers. In a recently published single-center ICU study from Türkiye, OXA-48-like enzymes were detected in 87.5%, *bla*_NDM_ in 70.8%, and *bla*_KPC_ in 12.5% of 96 carbapenem-resistant Enterobacterales isolates; 69.8% carried more than one carbapenemase gene, and *bla*_OXA-48_ and *bla*_NDM_ co-occurrence was the most frequent pattern. *bla*_VIM_ and *bla*_IMP_ were not detected in any isolate [20]. Although that study did not include *K. pneumoniae* exclusively and is therefore not directly comparable with ours, its findings support our observations regarding the predominance of *bla*_OXA-48_ and *bla*_NDM_, their co-occurrence, and the absence of *bla*_VIM_ and *bla*_IMP_. The same study used real-time PCR as the reference method and emphasized the importance of genotypic assessment for accurately identifying isolates carrying multiple carbapenemase genes [20].

The differences in molecular profiles and clinical case mix between the two centers could potentially confound the pooled gene–mortality associations. To reduce this potential confounding, study center was included as a covariate in all multivariable models. After adjustment for study center and the other measured confounders, no statistically significant associations between the molecular variables and 28-day ICU mortality were demonstrated; however, the estimates remained imprecise. The center effect observed in the exploratory Firth model should not be attributed solely to differences in resistance-gene distributions, as it may also reflect differences in patient characteristics, infection types, clinical practices, empirical antimicrobial policies, or local clonal epidemiology. Because of the limited sample size and sparse center-specific molecular subgroups, center-by-gene profile interactions and reliable center-stratified models could not be evaluated. Therefore, the molecular estimates represent adjusted average associations across the two-center cohort but cannot determine whether gene–mortality relationships were consistent between the centers. The observed molecular diversity nevertheless underscores the importance of local surveillance. This study has several strengths. Patients were prospectively enrolled and isolates were prospectively collected at two centers, consecutive ICU patients meeting the eligibility criteria were included, and each patient was represented by only the first clinical isolate. Positive cultures were not automatically classified as infections; culture-positive cases that did not meet the clinical infection criteria were excluded. Evaluating bloodstream infection, urinary tract infection, and ventilator-associated pneumonia within the same study population provided data on a broader ICU population than studies limited to patients with bacteremia. Other strengths included following patients until the end of their ICU stay, the absence of missing outcome data, and evaluating individual genes, gene number, and gene combinations using both unadjusted and multivariable analyses.

Firth penalized regression was also used to mitigate small-sample bias in the exploratory profile analysis. These findings should be interpreted in light of several limitations. The modest sample size and small molecular subgroups limited the precision of the estimates; therefore, clinically meaningful associations cannot be excluded despite the use of Firth penalization in the exploratory profile model. The two-center design and limited study period may also restrict generalizability. Acute disease severity, including severity at ICU admission, and antimicrobial treatment were not systematically captured across the cohort, leaving potential residual confounding despite adjustment for the measured covariates. Between-center heterogeneity may also persist after adjustment, and the sample size did not permit reliable center-stratified analyses or assessment of center-by-profile interactions. Molecular testing was limited to the targeted gene families without amplicon sequencing; subtypes, clonal relatedness, plasmid context, off-panel resistance mechanisms, and virulence determinants were not characterized. Such unresolved heterogeneity may have obscured subtype- or lineage-specific associations, which future whole-genome sequencing studies could help clarify. Finally, the outcomes reflected all-cause ICU mortality rather than infection-attributable mortality, and deaths after ICU discharge were not assessed. The findings should therefore be interpreted as exploratory associations rather than causal effects.

## 4. Materials and Methods

### 4.1. Study Design and Population

This prospective, multicenter, observational cohort study was conducted between April and September 2026 in the medical microbiology laboratories of Kahramanmaraş Necip Fazıl City Hospital and Van Training and Research Hospital. CRKP strains isolated from blood, urine, and tracheal aspirate specimens submitted to the laboratories as part of the routine diagnostic evaluation of ICU patients were assessed. Consecutive ICU patients aged 18 years or older from whom CRKP was isolated and who met the clinical infection criteria during the study period were included. No patients were selected on the basis of sampling or investigator discretion. Demographic and clinical patient data and microbiological isolate data were recorded prospectively. No patient was excluded from the analysis because of missing clinical data or loss to follow-up.

Each patient was included only once. If CRKP was isolated more than once from the same patient, the first isolate considered to be the cause of clinical infection was designated the index isolate. Subsequent isolates from the same patient were considered duplicates and excluded from the analysis regardless of specimen or infection type.

A positive culture alone was not considered evidence of infection. Infection status was determined by reviewing the clinical, laboratory, and, where applicable, radiological findings documented in the patients’ medical records at the time of culture. Bloodstream infection was defined as CRKP growth in at least one blood culture accompanied by fever, leukocytosis, elevated C-reactive protein, and other systemic findings compatible with infection. Urinary tract infection was defined as CRKP growth of ≥10^5^ CFU/mL in urine culture accompanied by compatible clinical and urinalysis findings. In non-intubated patients who were able to report symptoms, dysuria, urinary frequency, urgency, and suprapubic or costovertebral angle tenderness were evaluated. Urinalysis findings, including pyuria, leukocyte esterase positivity, and nitrite positivity, were assessed together with the clinical findings. In patients who were unable to report urinary symptoms, classification was based on the objective clinical, laboratory, and urinalysis findings documented in their medical records. Ventilator-associated pneumonia was defined as CRKP growth from a tracheal aspirate obtained after at least 48 h of invasive mechanical ventilation, together with compatible clinical findings and a new or progressive pulmonary infiltrate. Tracheal aspirate quality was assessed using the Gram-stain-based Q-score system, and specimens classified as Q2 or Q3 were considered acceptable for culture interpretation. Culture-positive cases that did not meet the corresponding clinical infection criteria were considered colonization or clinically insignificant growth and were excluded from the study. Each patient was represented by a single index isolate in the analyses, and the patient was defined as the unit of analysis. The date on which the index isolate was detected was considered the index date.

### 4.2. Patients, Bacterial Identification and Antimicrobial Susceptibility Testing

Blood and tracheal aspirate specimens were inoculated onto 5% sheep blood agar (SBA) (Becton, Dickinson and Company, Sparks, MD, USA), chocolate agar (Becton, Dickinson and Company, Sparks, MD, USA), and eosin methylene blue (EMB) agar (Becton, Dickinson and Company, Sparks, MD, USA), whereas urine specimens were inoculated onto 5% SBA and EMB agar. All plates were incubated at 37 °C for 18–24 h. Isolates were identified and antimicrobial susceptibility testing was performed using the VITEK^®^ 2 Compact system (bioMérieux, Marcy-l’Étoile, France). Results were interpreted according to the European Committee on Antimicrobial Susceptibility Testing (EUCAST) Clinical Breakpoint Tables, version 16.0 (2026) [21].

Ertapenem (10 µg), imipenem (10 µg), and meropenem (10 µg) (Becton, Dickinson and Company, Sparks, MD, USA) were tested to assess carbapenem susceptibility. Isolates classified as resistant to at least one of these carbapenems by the VITEK^®^ 2 Compact system underwent further evaluation, and the resistance phenotype was confirmed by disk diffusion using disks containing the same agents. Inhibition-zone diameters were interpreted according to the EUCAST Clinical Breakpoint Tables, version 16.0 (2026) [21]. *Escherichia coli* ATCC 25922 was used for routine disk diffusion quality control, while the KPC-producing *Klebsiella pneumoniae* ATCC BAA-1705 strain was included as a carbapenem-resistant positive control. All 94 isolates included in the study were resistant to ertapenem, imipenem, and meropenem by both methods, with no discordance in resistance categories between methods. Isolates were stored in stock medium at −80 °C until molecular analysis.

### 4.3. Molecular Detection of Resistance Genes

CRKP isolates were thawed from stock cultures stored at −80 °C, subcultured on 5% SBA, and incubated at 35 ± 2 °C for 24 h. Genomic DNA was extracted according to the manufacturer’s instructions using the magnetic bead-based Bio-Speedy^®^ Fast Nucleic Acid Extraction Kit (Bioeksen, İstanbul, Türkiye) and the Zybio EXM3000 automated nucleic acid extraction system (Zybio Inc., Chongqing, China). DNA samples were used for real-time PCR analysis immediately after extraction.

The carbapenemase genes *blaKPC*, *bla*_NDM_, *bla*_VIM_, *bla*_IMP_, and *bla*_OXA-48_ and the extended-spectrum beta-lactamase gene *bla*_CTX-M_ were investigated by multiplex real-time PCR using the Bio-Speedy^®^ Carbapenem Resistance PCR Kit (Bioeksen, İstanbul, Türkiye), which simultaneously detects these targets. Amplification reactions were prepared according to the manufacturer’s protocol and performed on the Mic qPCR System (Bio Molecular Systems, Upper Coomera, Australia). The kit’s positive, negative, and internal controls were included in each run. Amplification curves and cycle-threshold values were evaluated together, and the results were classified as positive or negative according to the manufacturer’s interpretation algorithm. The assay provided qualitative detection of the targeted *bla*_KPC_, *bla*_NDM_, *bla*_VIM_, *bla*_IMP_, *bla*_OXA-48_, and *bla*_CTX-M_ gene families. Allele-level variants or subtypes within these gene families were not differentiated, and the PCR amplicons were not subjected to sequencing. Resistance genes outside the targets included in the PCR panel were therefore not evaluated.

A carbapenemase profile was defined as the combination of carbapenemase genes detected in an isolate. Isolates carrying two or more carbapenemase genes were classified as carrying multiple carbapenemase genes. Because *bla*_CTX-M_ is not a carbapenemase gene, it was not included in the carbapenemase gene count or profile classification and was evaluated separately.

### 4.4. Clinical Data Collection and Outcomes

The age-adjusted CCI was calculated using the combined age–comorbidity index described by Charlson et al. [22]. Patient age, sex, study center, specimen type, and infection type were also recorded. Total ICU length of stay, time from ICU admission to detection of the index isolate, and time from the index culture to the end of the ICU stay were calculated in days.

The primary outcome was 28-day ICU mortality, defined as death occurring in the ICU within 28 days after the index date. Fourteen-day ICU mortality, defined as death occurring in the ICU within 14 days after the index date, and final ICU mortality were evaluated as secondary outcomes. Patients discharged alive from the ICU before day 14 or day 28 were classified as not having experienced the corresponding ICU mortality outcome. Final ICU mortality was defined as death occurring in the ICU at any time after the index date, without a specified time restriction. Patients were followed prospectively until ICU discharge or death; mortality occurring after ICU discharge was not assessed.

### 4.5. Statistical Analysis

Continuous variables are presented as medians and interquartile ranges, and categorical variables as numbers and percentages. Comparisons according to final ICU outcome were performed using the Mann-Whitney U test for continuous variables and Pearson’s chi-square test for categorical variables. All tests were two-sided, and *p* < 0.05 was considered statistically significant. No formal a priori power analysis was performed. The sample size was determined by the inclusion of all consecutive patients who met the eligibility criteria during the prespecified study period, and the precision of the estimates was evaluated using 95% confidence intervals. Because no data were missing for the variables used in the analyses, no imputation was performed, and all analyses included 94 patients.

The number of carbapenemase genes was calculated using *bla*_KPC_, *bla*_NDM_, *bla*_VIM_, *bla*_IMP_, and *bla*_OXA-48_. Isolates were classified as carrying one or two carbapenemase genes. The mutually exclusive profiles were defined as *bla*_KPC_ alone, *bla*_NDM_ alone, *bla*_OXA-48_ alone, and *bla*_NDM_ + *bla*_OXA-48_. *bla*_CTX-M_ was not included in the carbapenemase gene count; its presence and co-occurrence with carbapenemase genes were evaluated separately.

The unadjusted associations between the presence of *bla*_KPC_, *bla*_NDM_, *bla*_OXA-48_, or *bla*_CTX-M_ and 14-day, 28-day, and final ICU mortality were evaluated separately. *bla*_VIM_ and *bla*_IMP_ were excluded from association analyses because they were not detected in any isolate. Two-sided Fisher’s exact tests were used for binary comparisons, and the Fisher-Freeman-Halton exact test was used for the overall comparison of the four carbapenemase profiles [23]. Results are presented as unadjusted odds ratios and 95% confidence intervals. The *bla*_OXA-48_-alone group served as the reference in profile analyses. Molecular comparisons involving different resistance genes, carbapenemase gene number, and carbapenemase profiles were considered hypothesis-generating. No overall multiple-testing correction was applied to these analyses; results were interpreted on the basis of the magnitude of the odds ratios and their 95% confidence intervals, as well as the *p* values.

Multivariable binary logistic regression was used to evaluate factors associated with 28-day ICU mortality after adjustment for measured confounders. Models were adjusted for age-adjusted CCI, infection type, and study center. Age was not entered separately because it is a component of the age-adjusted index. Because specimen type and infection type overlapped completely, only infection type was included in the models.

The presence of *bla*_KPC_, *bla*_NDM_, *bla*_OXA-48_, and *bla*_CTX-M_ and the presence of two carbapenemase genes were evaluated in separate multivariable logistic regression models because these variables overlapped. Regression coefficients in these models were estimated using standard maximum likelihood; 95% confidence intervals and *p* values for the adjusted odds ratios were obtained using the Wald method. The linear relationship between CCI and the logit was assessed by adding a quadratic CCI term to the model and comparing the nested models using a likelihood-ratio test; no significant evidence of nonlinearity was demonstrated (*p* = 0.181).

Because the carbapenemase profile model contained small subgroups and the number of estimated parameters was high relative to the sample size, this analysis was considered exploratory. To reduce small-sample bias, the profile-based multivariable analysis used Firth’s bias-reduced penalized logistic regression based on the Jeffreys prior [24]. The *bla*_OXA-48_-alone profile was used as the reference category. Results are presented as Firth-adjusted odds ratios and 95% profile penalized-likelihood confidence intervals. The overall effect of the profile variable was evaluated using a penalized likelihood-ratio test. A sensitivity analysis using standard maximum likelihood estimation yielded results similar to those of the Firth model.

In exploratory between-center comparisons, continuous variables were evaluated using the Mann-Whitney U test, binary categorical variables using Fisher’s exact test, and variables with more than two categories using the Fisher-Freeman-Halton exact test. For the Fisher-Freeman-Halton test, all possible tables with fixed margins were enumerated exactly to calculate the two-sided exact *p* value; no Monte Carlo approximation was used. No multiple-testing correction was applied to these descriptive comparisons. Results are presented in Supplementary Table S1. Firth penalized logistic regression was implemented using custom Python code based on NumPy version 2.3.5 and SciPy version 1.17.0. All analyses were performed using Python version 3.12.13, pandas version 2.2.3, NumPy version 2.3.5, and SciPy version 1.17.0.

## 5. Conclusions

In conclusion, although OXA-48 was the predominant carbapenemase in this two-center ICU cohort, *bla*_NDM_ and *bla*_OXA-48_-*bla*_NDM_ co-occurrence were also common, and *bla*_CTX-M_ frequently co-occurred with carbapenemase genes. No statistically significant associations were demonstrated between early or final ICU mortality and the individual presence of the investigated genes, the presence of two carbapenemase genes, or the identified gene profiles. However, clinically meaningful associations cannot be excluded because of the uncertainty surrounding the estimates. Among the measured clinical variables, comorbidity burden and infection type were associated with 28-day ICU mortality. These findings indicate that molecular resistance characteristics remain important for epidemiological surveillance and that gene-mortality associations should be evaluated in larger samples together with acute disease-severity and treatment variables. Larger multicenter studies supported by genomic analyses may more precisely define the relationship between carbapenemase types and clinical outcomes.

## Figures and Tables

**Figure 1:**
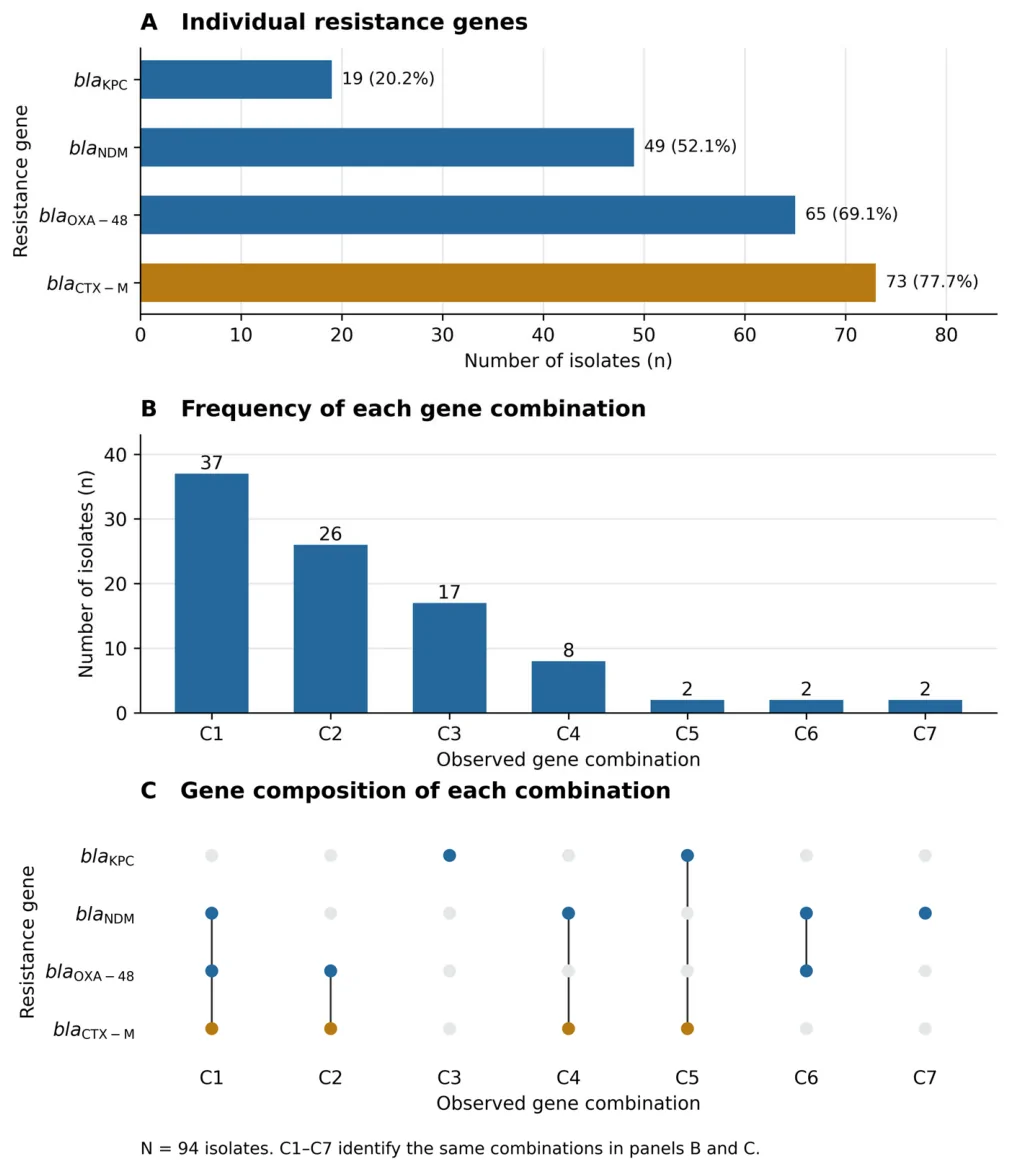
Distribution and co-occurrence of resistance genes among 94 CRKP isolates. (**A**) Number and percentage of isolates positive for each detected gene; gene frequencies are not mutually exclusive. The *x*-axis shows the number of isolates and the *y*-axis identifies the resistance gene. (**B**) Number of isolates with each mutually exclusive observed gene combination. The *x*-axis identifies combinations C1–C7 and the *y*-axis shows the number of isolates. (**C**) Composition of combinations C1–C7, aligned with panel B. Colored dots indicate detected genes, gray dots indicate genes not detected within the combination, and connecting lines link genes co-occurring in the same isolate. Carbapenemase genes are shown in blue and *bla*_CTX-M_, an ESBL gene, in gold. *bla*_VIM_ and *bla*_IMP_ were not detected in any isolate. CRKP, carbapenem-resistant *Klebsiella pneumoniae*; ESBL, extended-spectrum beta-lactamase.

**Figure 2:**
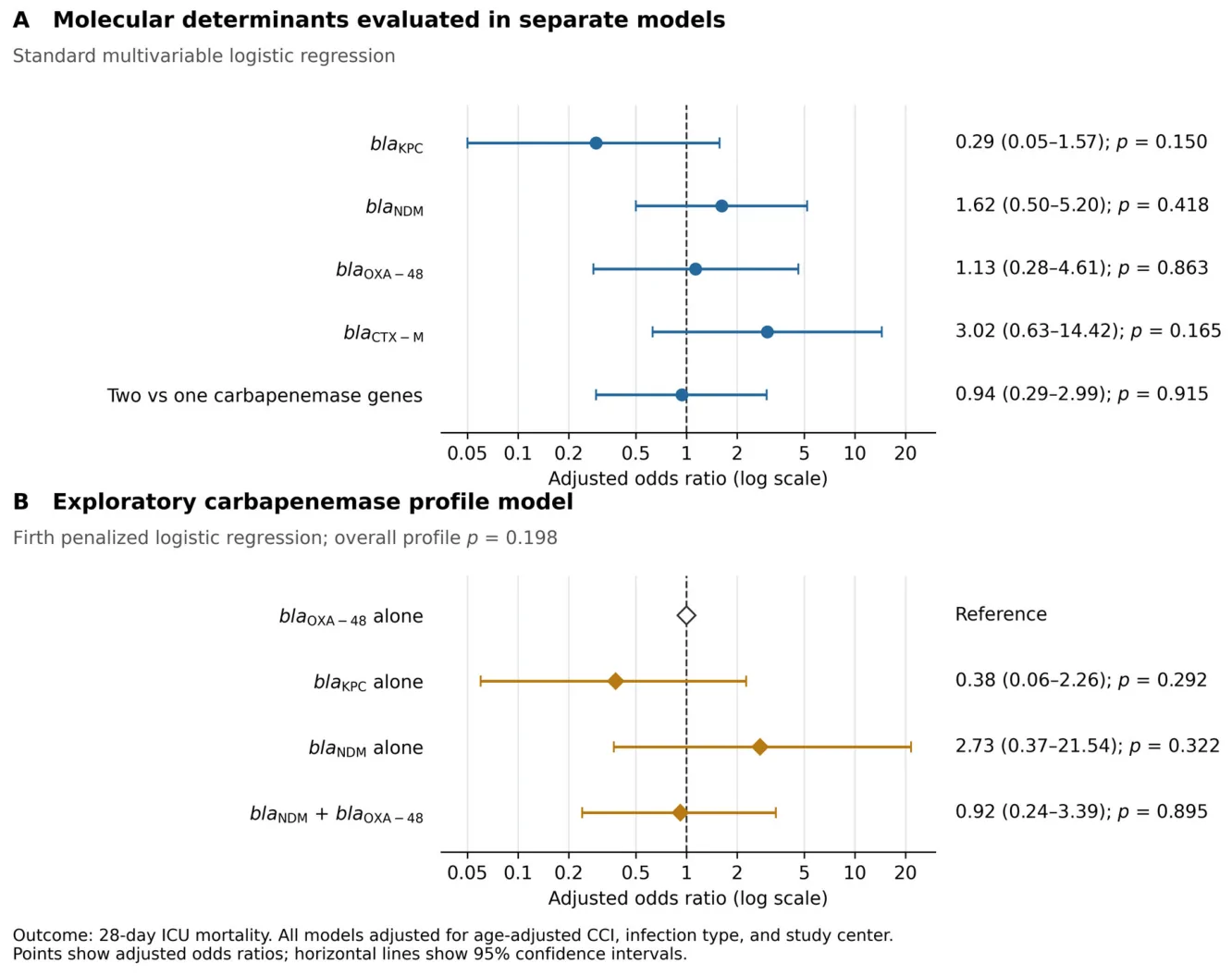
Adjusted associations between molecular resistance characteristics and 28-day ICU mortality. (**A**) Individual gene presence versus absence and two versus one carbapenemase genes were evaluated in separate standard multivariable logistic regression models. (**B**) Mutually exclusive carbapenemase profiles were evaluated using exploratory Firth penalized logistic regression, with *bla*_OXA-48_ alone as the reference. All models were adjusted for age-adjusted Charlson Comorbidity Index, infection type, and study center. The *x*-axis shows adjusted odds ratios on a logarithmic scale and the *y*-axis identifies the molecular variables or profiles. Points indicate adjusted odds ratios and horizontal lines indicate 95% confidence intervals (Wald intervals in panel (**A**) and profile penalized-likelihood intervals in panel (**B**)). The open diamond identifies the reference group; the dashed vertical line denotes an odds ratio of 1. The overall profile association was evaluated using a penalized likelihood-ratio test (*p* = 0.198). Numerical annotations show adjusted odds ratios, 95% confidence intervals, and *p* values. ICU, intensive care unit.

**Table 1:** Demographic and clinical characteristics of patients according to ICU outcome.

Characteristics	Overall (*n* = 94)	Survivors (*n* = 41)	Non-Survivors (*n* = 53)	*p* Value
Age, years	70 (58–78)	67 (57–75)	71 (60–81)	0.056
Age-adjusted Charlson Comorbidity Index	6 (4–8)	5 (3–6)	7 (6–9)	**<0.001**
**Sex**				0.164
Female	35 (37.2)	19 (46.3)	16 (30.2)	
Male	59 (62.8)	22 (53.7)	37 (69.8)	
**Study center**				0.716
Van Training and Research Hospital	49 (52.1)	20 (48.8)	29 (54.7)	
Necip Fazıl City Hospital	45 (47.9)	21 (51.2)	24 (45.3)	
**Specimen and infection type**				**0.010**
Blood—bloodstream infection	30 (31.9)	8 (19.5)	22 (41.5)	
Tracheal aspirate—ventilator-associated pneumonia	33 (35.1)	13 (31.7)	20 (37.7)	
Urine—urinary tract infection	31 (33.0)	20 (48.8)	11 (20.8)	
Total ICU length of stay, days	22 (10–58)	24 (8–80)	21 (10–42)	0.401
Time from ICU admission to the first CRKP-positive culture, days	7 (1–20)	7 (1–16)	7 (2–21)	0.321
ICU length of stay after the first CRKP-positive culture, days	10 (5–25)	14 (6–45)	8 (5–17)	**0.011**

Data are presented as median (interquartile range) or *n* (%). Percentages were calculated within columns. Continuous variables were compared using the Mann–Whitney U test, and categorical variables were compared using Pearson’s chi-square test with Yates’ continuity correction for 2 × 2 tables. All tests were two-sided. Bold *p* values indicate statistical significance (*p* < 0.05). ICU, intensive care unit; CRKP, carbapenem-resistant *Klebsiella pneumoniae*.

**Table 2:** Distribution of resistance genes and carbapenemase gene profiles among CRKP isolates.

Molecular Characteristic	*n* (%)
**Individual resistance genes**	
*bla*_KPC_	19 (20.2)
*bla*_NDM_	49 (52.1)
*bla*_VIM_	0 (0.0)
*bla*_IMP_	0 (0.0)
*bla*_OXA-48_	65 (69.1)
*bla*_CTX-M_	73 (77.7)
**Number of carbapenemase genes**	
One carbapenemase gene	55 (58.5)
Two carbapenemase genes	39 (41.5)
**Mutually exclusive carbapenemase gene profiles**	
*bla*_KPC_ alone	19 (20.2)
*bla*_NDM_ alone	10 (10.6)
*bla*_OXA-48_ alone	26 (27.7)
*bla*_NDM_ + *bla*_OXA-48_	39 (41.5)
**Complete resistance-gene profiles including** ***bla*** **_CTX-M_**	
*bla*_KPC_	17 (18.1)
*bla*_KPC_ + *bla*_CTX-M_	2 (2.1)
*bla*_NDM_	2 (2.1)
*bla*_NDM_ + *bla*_CTX-M_	8 (8.5)
*bla*_OXA-48_ + *bla*_CTX-M_	26 (27.7)
*bla*_NDM_ + *bla*_OXA-48_	2 (2.1)
*bla*_NDM_ + *bla*_OXA-48_ + *bla*_CTX-M_	37 (39.4)

Data are presented as *n* (%). Percentages were calculated using the total number of isolates (*n* = 94) as the denominator. Individual gene frequencies are not mutually exclusive. Carbapenemase gene number and profiles were defined using *bla*_KPC_, *bla*_NDM_, *bla*_VIM_, *bla*_IMP_, *bla*_OXA-48_, and *bla*_CTX-M_ was not included in the carbapenemase gene count because it encodes an extended-spectrum β-lactamase. Each patient contributed only the first CRKP isolate. Note: *bla*_NDM_ + *bla*_OXA-48_ in the “Mutually exclusive carbapenemase profiles” section (*n* = 39) is defined irrespective of *bla*_CTX-M_ status; *bla*_NDM_ + *bla*_OXA-48_ in the “Complete resistance-gene profiles including *bla*_CTX-M_” section (*n* = 2) refers only to the *bla*_CTX-M_-negative subgroup. CRKP, carbapenem-resistant *Klebsiella pneumoniae.*

**Table 3:** Associations of individual resistance genes and carbapenemase gene number with ICU mortality.

Molecular Characteristic	14-Day ICU Mortality, *n*/N (%)	OR (95% CI)	*p* Value	28-Day ICU Mortality, *n*/N (%)	OR (95% CI)	*p* Value	Final ICU Mortality, n/N (%)	OR (95% CI)	*p* Value
*bla*_KPC_ positive	5/19 (26.3)	0.45 (0.15–1.39)	0.197	9/19 (47.4)	0.83 (0.30–2.28)	0.800	12/19 (63.2)	1.42 (0.50–4.01)	0.608
*bla*_NDM_ positive	22/49 (44.9)	1.48 (0.64–3.39)	0.404	27/49 (55.1)	1.40 (0.62–3.16)	0.536	28/49 (57.1)	1.07 (0.47–2.41)	1.000
*bla*_OXA-48_ positive	30/65 (46.2)	2.25 (0.87–5.81)	0.113	33/65 (50.8)	0.96 (0.40–2.31)	1.000	35/65 (53.8)	0.71 (0.29–1.74)	0.506
*bla*_CTX-M_ positive	33/73 (45.2)	2.64 (0.87–7.97)	0.129	38/73 (52.1)	1.19 (0.45–3.16)	0.807	40/73 (54.8)	0.75 (0.28–2.02)	0.624
Two carbapenemase genes	19/39 (48.7)	1.80 (0.78–4.16)	0.203	21/39 (53.8)	1.21 (0.53–2.75)	0.680	22/39 (56.4)	1.00 (0.44–2.29)	1.000
One carbapenemase gene	19/55 (34.5)	Reference		27/55 (49.1)	Reference		31/55 (56.4)	Reference	

Mortality percentages were calculated within each molecular group. Odds ratios are unadjusted. For individual gene analyses, patients negative for the corresponding gene constituted the reference group. For the carbapenemase gene-number analysis, isolates carrying one carbapenemase gene constituted the reference group. Two-sided Fisher’s exact tests were used for binary comparisons. *bla*_VIM_ and *bla*_IMP_ were excluded because no positive isolates neither gene was detected in any isolate. CI, confidence interval; ICU, intensive care unit; OR, odds ratio.

**Table 4:** Associations of mutually exclusive carbapenemase gene profiles with ICU mortality.

Carbapenemase Gene Profile	14-Day ICU Mortality, *n*/N (%)	OR (95% CI)	*p* Value	28-Day ICU Mortality, *n*/N (%)	OR (95% CI)	*p* Value	Final ICU Mortality, n/N (%)	OR (95% CI)	*p* Value
*bla*_KPC_ alone	5/19 (26.3)	0.49 (0.13–1.76)	0.351	9/19 (47.4)	1.05 (0.32–3.44)	1.000	12/19 (63.2)	1.71 (0.51–5.74)	0.545
*bla*_NDM_ alone	3/10 (30.0)	0.58 (0.12–2.78)	0.706	6/10 (60.0)	1.75 (0.40–7.70)	0.711	6/10 (60.0)	1.50 (0.34–6.59)	0.717
*bla*_OXA-48_ alone	11/26 (42.3)	Reference		12/26 (46.2)	Reference		13/26 (50.0)	Reference	
*bla*_NDM_ + *bla*_OXA-48_	19/39 (48.7)	1.30 (0.48–3.52)	0.800	21/39 (53.8)	1.36 (0.50–3.68)	0.617	22/39 (56.4)	1.29 (0.48–3.50)	0.623
Overall profile comparison			0.379			0.878			0.845

Mortality percentages were calculated within each molecular group. Odds ratios are unadjusted, with 95% Wald confidence intervals. The *bla*_OXA-48_-alone group was the reference category for pairwise comparisons, and pairwise *p* values were calculated using two-sided Fisher’s exact tests. Overall comparisons among the four mutually exclusive carbapenemase profiles were performed using the two-sided Fisher–Freeman–Halton exact test with exhaustive enumeration: 14-day ICU mortality, *p* = 0.379; 28-day ICU mortality, *p* = 0.878; final ICU mortality, *p* = 0.845. CI, confidence interval; ICU, intensive care unit; OR, odds ratio.

**Table 5:** Multivariable logistic regression analysis of factors associated with 28-day ICU mortality.

Variable	Adjusted OR	95% CI	*p* Value
**A. Molecular determinants evaluated in separate models**			
*bla*_KPC_ present vs. absent	0.29	0.05–1.57	0.150
*bla*_NDM_ present vs. absent	1.62	0.50–5.20	0.418
*bla*_OXA-48_ present vs. absent	1.13	0.28–4.61	0.863
*bla*_CTX-M_ present vs. absent	3.02	0.63–14.42	0.165
Two carbapenemase genes vs. one gene	0.94	0.29–2.99	0.915
**B. Exploratory Firth penalized carbapenemase profile model**			0.198 ᵃ
*bla*_OXA-48_ alone	Reference	—	—
*bla*_KPC_ alone	0.38	0.06–2.26	0.292
*bla*_NDM_ alone	2.73	0.37–21.54	0.322
*bla*_NDM_ + *bla*_OXA-48_	0.92	0.24–3.39	0.895
Age-adjusted CCI, per 1-point increase	1.91	1.47–2.67	<0.001
Infection type			
Urinary tract infection	Reference	—	—
Bloodstream infection	13.10	3.00–72.58	<0.001
Ventilator-associated pneumonia	5.16	1.31–24.25	0.018
Study center			
Van Training and Research Hospital	Reference	—	—
Necip Fazıl City Hospital	0.29	0.08–0.99	0.048

OR, odds ratio; CI, confidence interval; CCI, Charlson Comorbidity Index; KPC, *Klebsiella pneumoniae* carbapenemase; NDM, New Delhi metallo-β-lactamase. In Panel A, each molecular determinant was evaluated in a separate standard maximum-likelihood logistic regression model adjusted for age-adjusted CCI, infection type, and study center. Panel B presents an exploratory Firth bias-reduced penalized logistic regression model containing mutually exclusive carbapenemase profiles and the same adjustment variables; estimates are Firth-adjusted ORs with 95% profile penalized-likelihood CIs. CTX-M was not included in the carbapenemase gene count. Because specimen type and infection type overlapped completely, only infection type was included in the models. ᵃ Penalized likelihood-ratio test for the overall profile variable.

## Data Availability

The de-identified data supporting the findings of this study are available from the corresponding author upon reasonable request. The data are not publicly available because they contain sensitive clinical information and their public sharing was not covered by the informed consent and ethics approval.

## Supplementary Materials

The following supporting information can be downloaded at https://www.mdpi.com/article/10.3390/ijms27209388/s1.

## Abbreviations

The following abbreviations are used in this manuscript:

CCI	Charlson Comorbidity Index
CFU	colony-forming unit
CI	confidence interval
CRE	carbapenem-resistant Enterobacterales
CRKP	carbapenem-resistant *Klebsiella pneumoniae*
CTX-M	cefotaximase-Munich
DNA	deoxyribonucleic acid
ESBL	extended-spectrum beta-lactamase
EUCAST	European Committee on Antimicrobial Susceptibility Testing
ICU	intensive care unit
IMP	imipenemase
IQR	interquartile range
KPC	*Klebsiella pneumoniae* carbapenemase
NDM	New Delhi metallo-β-lactamase
OR	odds ratio
OXA-48	oxacillinase-48
PCR	polymerase chain reaction
SBA	Sheep Blood agar
SOFA	Sequential Organ Failure Assessment
STROBE	Strengthening the Reporting of Observational Studies in Epidemiology
VIM	Verona integron-encoded metallo-β-lactamase
WHO	World Health Organization
