# Supplementary material for: Beyond Resistance: Carbapenemase Gene Profiles and Mortality in ICU Patients with Carbapenem-Resistant Klebsiella pneumoniae Infections
Source: Int J Mol Sci. 2026 Oct 21;27(20):9388. doi: 10.3390/ijms27209388 (PMC13704951; doi:10.3390/ijms27209388)
Supplement: Supplementary file 1 [file ijms-27-09388-s001.zip › STROBE_Checklist_CRKP.pdf]

STROBE Checklist for Cohort Studies

Beyond Resistance: Carbapenemase Gene Profiles and Mortality in ICU Patients with Carbapenem-Resistant Klebsiella pneumoniae Infections

Section/topic	Item	Recommendation	Reported on page/section
**Title and abstract**	1(a)	Indicate the study's design with a commonly used term in the title or the abstract	Abstract, p. 2 (prospective two-center cohort)
	1(b)	Provide in the abstract an informative and balanced summary of what was done and what was found	Abstract, p. 2
**Introduction**			
**Background/rationale**	2	Explain the scientific background and rationale for the investigation being reported	Introduction, pp. 2-3
**Objectives**	3	State specific objectives, including any prespecified hypotheses	Introduction, p. 3
**Methods**			
**Study design**	4	Present key elements of study design early in the paper	Section 4.1, p. 14
**Setting**	5	Describe the setting, locations, and relevant dates, including periods of recruitment, exposure, follow-up, and data collection	Sections 4.1 and 4.4, pp. 14-16
**Participants**	6(a)	Give the eligibility criteria, and the sources and methods of selection of participants. Describe methods of follow-up	Sections 4.1 and 4.4, pp. 14-16
	6(b)	For matched studies, give matching criteria and number of exposed and unexposed	Not applicable; this was not a matched study
**Variables**	7	Clearly define all outcomes, exposures, predictors, potential confounders, and effect modifiers. Give diagnostic criteria, if applicable	Sections 4.1, 4.3 and 4.4, pp. 14-16
**Data sources/ measurement**	8*	For each variable of interest, give sources of data and details of methods of assessment (measurement). Describe comparability of assessment methods if there is more than one group	Sections 4.1-4.4, pp. 14-16
**Bias**	9	Describe any efforts to address potential sources of bias	Section 4.1, p. 14; Section 4.5, pp. 16-17; limitations, pp. 13-14
**Study size**	10	Explain how the study size was arrived at	Section 4.5, p. 16
**Quantitative variables**	11	Explain how quantitative variables were handled in the analyses. If applicable, describe which groupings were chosen and why	Sections 4.4-4.5, pp. 15-16
**Statistical methods**	12(a)	Describe all statistical methods, including those used to control for confounding	Section 4.5, pp. 16-17
	12(b)	Describe any methods used to examine subgroups and interactions	Exploratory between-center comparisons, Section 4.5, p. 17; no interaction analyses were performed
	12(c)	Explain how missing data were addressed	Section 4.5, p. 16 (no missing analysis data; no imputation)
	12(d)	If applicable, explain how loss to follow-up was addressed	Sections 4.1 and 4.4, pp. 14 and 16 (no loss to follow-up)
	12(e)	Describe any sensitivity analyses	Section 4.5, p. 17
**Results**			

Section/topic	Item	Recommendation	Reported on page/section
**Participants**	13(a)*	Report numbers of individuals at each stage of study - e.g., numbers potentially eligible, examined for eligibility, confirmed eligible, included in the study, completing follow-up, and analysed	Section 2.1, p. 3; Sections 4.1 and 4.5, pp. 14 and 16. A total of 94 patients were included, completed follow-up, and were analysed
	13(b)*	Give reasons for non-participation at each stage	Section 4.1, p. 14 (duplicate isolates and culture-positive cases not meeting clinical infection criteria were excluded); stage-specific counts were not retained
	13(c)*	Consider use of a flow diagram	Not provided; stage-specific screening and exclusion counts were not retained
**Descriptive data**	14(a)*	Give characteristics of study participants (e.g., demographic, clinical, social) and information on exposures and potential confounders	Section 2.1 and Table 1, pp. 3-4; Section 2.2 and Table 2, pp. 5-6
	14(b)*	Indicate number of participants with missing data for each variable of interest	Section 4.5, p. 16 (no missing data in analysis variables)
	14(c)*	Summarise follow-up time (e.g., average and total amount)	Table 1, p. 4; Section 4.4, pp. 15-16
**Outcome data**	15*	Report numbers of outcome events or summary measures over time	Abstract, p. 2; Section 2.1, p. 3; Table 3, pp. 7-8
**Main results**	16(a)	Give unadjusted estimates and, if applicable, confounder-adjusted estimates and their precision (e.g., 95% confidence interval). Make clear which confounders were adjusted for and why they were included	Sections 2.3-2.4 and Tables 3-4, pp. 6-9; Section 4.5, p. 16
	16(b)	Report category boundaries when continuous variables were categorized	Not applicable to the continuous covariate; CCI was modelled per one-point increase. Molecular group definitions are reported in Section 4.5, p. 16
	16(c)	If relevant, consider translating estimates of relative risk into absolute risk for a meaningful time period	Absolute mortality counts and percentages are reported in the Abstract and Section 2.1, pp. 2-3, and Table 3, pp. 7-8
**Other analyses**	17	Report other analyses done - e.g., analyses of subgroups and interactions, and sensitivity analyses	Sections 2.4-2.5, pp. 8-10; Supplementary Table S1; sensitivity analysis described in Sections 2.4 and 4.5, pp. 8 and 17
**Discussion**			
**Key results**	18	Summarise key results with reference to study objectives	Discussion, pp. 11-13; Conclusions, p. 17
**Limitations**	19	Discuss limitations of the study, taking into account sources of potential bias or imprecision. Discuss both direction and magnitude of any potential bias	Discussion, pp. 13-14
**Interpretation**	20	Give a cautious overall interpretation of results considering objectives, limitations, multiplicity of analyses, results from similar studies, and other relevant evidence	Discussion, pp. 11-14; Conclusions, p. 17
**Generalisability**	21	Discuss the generalisability (external validity) of the study results	Discussion, pp. 13-14; Conclusions, p. 17
**Other information**			
**Funding**	22	Give the source of funding and the role of the funders for the present study and, if applicable, for the original study on which the present article is based	Funding statement, p. 17

** Give information separately for exposed and unexposed groups where applicable.*

Source: STROBE Statement - Checklist of items that should be included in reports of cohort studies. Available at https://www.strobe-statement.org/checklists/
