# Supplementary material for: Beyond Resistance: Carbapenemase Gene Profiles and Mortality in ICU Patients with Carbapenem-Resistant Klebsiella pneumoniae Infections
Source: Int J Mol Sci. 2026 Oct 21;27(20):9388. doi: 10.3390/ijms27209388 (PMC13704951; doi:10.3390/ijms27209388)
Supplement: Supplementary file 1 [file ijms-27-09388-s001.zip › SuppTable1rev.pdf]

Supplementary Table S1. Clinical and molecular characteristics according to study center

Variable	Van Training and Research Hospital (*n* = 49)	Necip Fazıl City Hospital (*n* = 45)	*p* value
Age, years	64 (57–77)	74 (61–79)	0.092
Age-adjusted Charlson Comorbidity Index	5 (3–7)	7 (6–9)	**0.003**
Total ICU stay, days	23 (10–54)	21 (8–60)	0.859
ICU day of first positive culture, days	8 (2–18)	3 (1–20)	0.102
ICU stay after first positive culture, days	9 (3–25)	11 (5–20)	0.386
Female sex	14 (28.6)	21 (46.7)	0.089
14-day ICU mortality	24 (49.0)	14 (31.1)	0.095
28-day ICU mortality	28 (57.1)	20 (44.4)	0.302
Final ICU mortality	29 (59.2)	24 (53.3)	0.678
**Specimen/infection type**			**0.006**
Blood/bloodstream infection	20 (40.8)	10 (22.2)	
Urine/urinary tract infection	9 (18.4)	22 (48.9)	
Tracheal aspirate/ventilator-associated pneumonia	20 (40.8)	13 (28.9)	
**Resistance genes**			
KPC	5 (10.2)	14 (31.1)	**0.019**
NDM	28 (57.1)	21 (46.7)	0.409
OXA-48	43 (87.8)	22 (48.9)	**<0.001**
CTX-M	43 (87.8)	30 (66.7)	**0.024**
**Number of carbapenemase genes**			**0.007**
One carbapenemase gene	22 (44.9)	33 (73.3)	
Two carbapenemase genes	27 (55.1)	12 (26.7)	
**Carbapenemase profile**			**<0.001**
KPC alone	5 (10.2)	14 (31.1)	
NDM alone	1 (2.0)	9 (20.0)	
OXA-48 alone	16 (32.7)	10 (22.2)	
NDM + OXA-48	27 (55.1)	12 (26.7)	

Data are presented as median (interquartile range) or n (%). Continuous variables were compared using the Mann–Whitney U test. Binary categorical variables were compared using Fisher's exact test, and variables with more than two categories were compared using the Fisher–Freeman–Halton exact test. Percentages were

calculated within each study center. CTX-M was not included in the carbapenemase gene count. VIM and IMP could not be demonstrated in isolates from either center.
